# Quality of Patient Care in Critical Care Units: In Relation to Nurse/Patient Ratio

**DOI:** 10.1186/2197-425X-3-S1-A479

**Published:** 2015-10-01

**Authors:** A-C Falk, E-M Wallin

**Affiliations:** Karolinska University Hospital, CIVA, Solna, Sweden; Karolinska University Hospital, CIVA, Stockholm, Sweden

## Introduction

Intensive care is one of the most resource-intensive forms of medical care due to severely ill patients [[1]].

## Objectives

To measure optimal medical and nursing-related result usually used indicators is mortality, complications in intensive care, quality of life and functional status after intensive care. Among these indicators, only the frequency of VAP and multi-resistant bacteria that coincides with the Institute of Medicine (2001) scientifically approved nursing indicators for person-centered care. However none of these indicators is collected in the Swedish registry(SIR) therefore time on noninvasive and invasive ventilator treatment is collected to describe how indicators are a useful measure of Swedish intensive care from the nursing perspective is measured.

## Methods

This is a retrospective registry study includes a survey of critical care of registry data (all patients> 15 years) receiving care in six general Level I critical care units during 2010-2014. Data of nurse/patient ratio is collected from each unit. the data is analyzed by descriptive and comparative statistical methods.

## Results

Preliminary result shows an increase in critical care admissions from 7331 in 2011 to 7653 in the year 2014 throughout six university hospitals in Sweden. There was a large range in admissions between hospitals n= 667-2313. the result showed that 83% (5 out of 6) of the hospitals had a specialized nurse/patient ratio of 0.5-0.75 and one hospital had a 1:1 ratio. the hospitals care for adult patients between 17-100 years of age with 40% female and 60 percent are male patients with 30 days mortality of 18%. Complications during critical care was measured by readmission and unplanned reintubation and showed that unplanned reintubation was rare 2-8% with the highest rate at the hospital with the lowest rate of patients receiving invasive ventilation. Readmissions were found in 2-5% of all admissions, no correlation was found.

## Conclusions

There is an increase in critical care admissions throughout Sweden between the years 2010-2014. Preliminary results show differences in nurse/patient ratios in general critical care units throughout Sweden but data to measure optimal nurse/patient ratio is lacking. Further analysis is needed.
